# DUF1220 Dosage Is Linearly Associated with Increasing Severity of the Three Primary Symptoms of Autism

**DOI:** 10.1371/journal.pgen.1004241

**Published:** 2014-03-20

**Authors:** Jonathan M. Davis, Veronica B. Searles, Nathan Anderson, Jonathon Keeney, Laura Dumas, James M. Sikela

**Affiliations:** 1 Department of Biochemistry & Molecular Genetics, Human Medical Genetics and Genomics Program & Neuroscience Program, University of Colorado School of Medicine, Anschutz Medical Campus, Aurora, Colorado, United States of America; 2 Medical Scientist Training Program, University of Colorado School of Medicine, Anschutz Medical Campus, Aurora, Colorado, United States of America; Indiana University, United States of America

## Abstract

One of the three most frequently documented copy number variations associated with autism spectrum disorder (ASD) is a 1q21.1 duplication that encompasses sequences encoding DUF1220 protein domains, the dosage of which we previously implicated in increased human brain size. Further, individuals with ASD frequently display accelerated brain growth and a larger brain size that is also associated with increased symptom severity. Given these findings, we investigated the relationship between DUF1220 copy number and ASD severity, and here show that in individuals with ASD (n = 170), the copy number (dosage) of DUF1220 subtype CON1 is highly variable, ranging from 56 to 88 copies following a Gaussian distribution. More remarkably, in individuals with ASD CON1 copy number is also linearly associated, in a dose-response manner, with increased severity of each of the three primary symptoms of ASD: social deficits (p = 0.021), communicative impairments (p = 0.030), and repetitive behaviors (p = 0.047). These data indicate that DUF1220 protein domain (CON1) dosage has an ASD-wide effect and, as such, is likely to be a key component of a major pathway underlying ASD severity. Finally, these findings, by implicating the dosage of a previously unexamined, copy number polymorphic and brain evolution-related gene coding sequence in ASD severity, provide an important new direction for further research into the genetic factors underlying ASD.

## Author Summary

Autism Spectrum Disorder (ASD) is a common behaviorally defined condition noted by impairments in social reciprocity and communicative abilities and exaggerated repetitive behaviors and stereotyped interests. Individuals with ASD frequently have a larger and more rapidly growing brain than their typically developing peers. Given the widely documented heritability suggesting that ASD is predominantly a genetic condition and the well-established link between ASD and abnormal brain growth patterns, genes involved in brain growth would be excellent candidates to study regarding ASD. One such candidate is DUF1220, a highly copy number polymorphic protein domain that we have previously linked to brain evolution and brain size. However, due to the extreme copy number variability of DUF1220, it has not been directly investigated in previous genome wide polymorphism studies searching for genes important in ASD. Here we show that, in individuals with ASD, 1) DUF1220 subtype CON1 is highly variable, ranging from 56 to 88 copies, and 2) the copy number of CON1 is associated, in a linear dose-response manner, with increased severity of each of the three primary symptoms of ASD: as CON1 copy number increases each of the three primary symptoms of ASD (impaired social reciprocity, impaired communicative ability and increased repetitive behaviors) become incrementally worse.

## Introduction

Autism Spectrum Disorder (ASD) is a common neurodevelopmental condition characterized by impaired social reciprocity and communicative skills, as well as increased repetitive behaviors and stereotyped interests [1]. ASD has been frequently linked to an accelerated postnatal brain growth [2] that likely involves excessive neuron number and increased neuron density [3] which may affect symptom presentation through gray matter and total volumetric increases [4]–[6].

To date, despite the existence of a strong genetic component for ASD etiology [7], only rare- and minor-affect genetic loci have been identified [8], raising the possibility that major genetic contributors to ASD reside in previously unexplored parts of the genome. One such genomic candidate is DUF1220, a protein domain with an unusually broad spectrum of allelic copy number variation within the human population [9], [10]. Found within the *NBPF* gene family and primarily in the 1q21.1 region, DUF1220 sequences have undergone a rapid, recent and extreme increase in copy number specifically in the human lineage [11], [12]. Humans have approximately 290 haploid copies of DUF1220 that can be subdivided into 6 clades defined by sequence similarity (CON1-3 and HLS1-3) [12]. Further, DUF1220 copy number (dosage) has been implicated in normal and pathological variation in human brain size and in neuron number across primate lineages [10]. These findings, together with our recent research implicating DUF1220 domains as drivers of neuronal stem cell proliferation (J. Keeney, submitted), make DUF1220 an attractive candidate for modifying ASD symptoms through brain growth mechanisms. Finally, many DUF1220 domain paralogs reside in or adjacent to a widely documented 1q21.1 duplication that is one of the three most prevalent copy number variations (CNVs) significantly enriched in individuals with autism [13]–[15], lending further support to the link between DUF1220 copy number and ASD.

The association between DUF1220 copy number and the evolutionary expansion of the human brain [10], [15], [16], and the rapidity with which DUF1220 copy number increased in the human genome suggests there were strong selection pressures acting on these sequences [9]. We have suggested that this has also resulted in a deleterious genomic side effect: increased 1q21 instability that predisposes the region to deletions and duplications that in turn contribute to a large number of neurodevelopmental diseases including ASD [15]. This association of DUF1220 copy number increase with evolutionary adaptation may also help explain why ASD, which is genetic but maladaptive, has persisted at such a high frequency across human populations.

Given these insights and the link between the copy number of the CON1 subtype (clade) of DUF1220 domain and gray matter volume [10], along with the known associations between gray matter volume irregularities and ASD symptomology [6], we investigated the association between CON1 copy number and both parent-reported and clinically evaluated ASD-related symptoms. Phenotypic characteristics of children with ASD were determined by clinically robust metrics and CON1 copy numbers were determined using droplet digital PCR (ddPCR), a third-generation PCR technique designed for accurate assay of copy number measurement.

## Results

**Figure 1:**
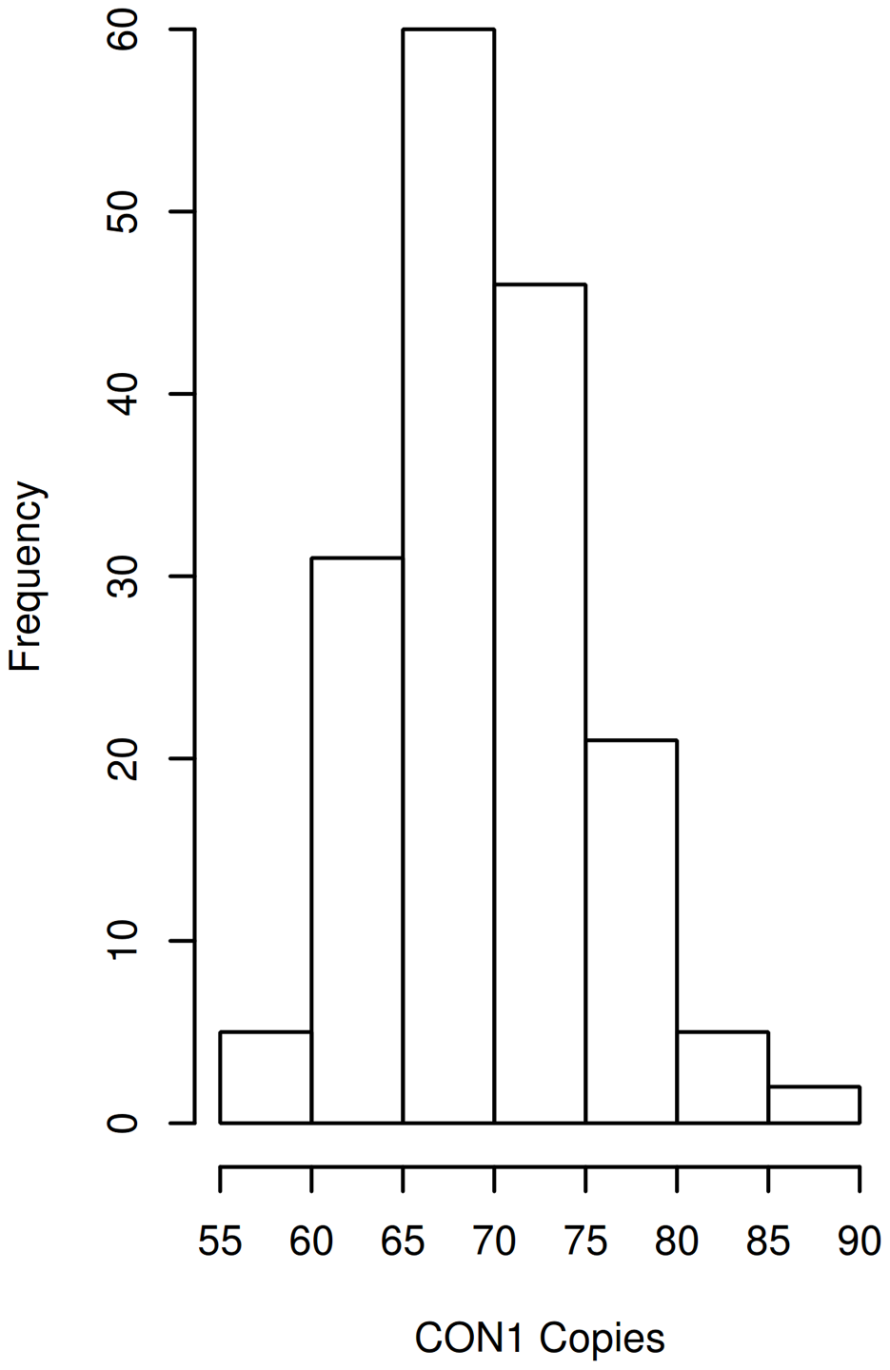
DUF1220 CON1 copy number distribution in individuals with ASD. CON1 copy numbers were determined for 170 individuals with ASD. CON1 copy number ranges are indicated. Frequency denotes the number of individuals who exhibited the indicated copy number range.

Notably, the CON1 copy number profile in individuals with ASD followed a Gaussian distribution (Figure 1). In ASD samples CON1 had a mean of 70 copies and extended from 56 to 88, a range that was similar to that found in otherwise healthy individuals (ASD mean = 70, SD = 5.5, healthy mean = 70, SD = 6.9, unequal variance ttest p = 0.98). However, multivariate linear regression detected a linear increase in CON1 dosage that was progressively associated with increasing severity of each of the three primary symptoms associated with ASD as measured by the ADI-R (Table 1). With each additional copy of CON1, Social Diagnostic Score increased on average 0.25 points (SE 0.11 p = 0.021), Communicative Diagnostic Score increased 0.18 points (SE 0.08 p = 0.030) and Repetitive Behavior Diagnostic Score increased 0.10 points (SE = 0.05 p = 0.047). Further, the association between CON1 copy number and Vineland Adaptive Behavior Scale (VABS)-measured Standardized Social Score was nearly significant (p = 0.057), also indicating a progressively worsening condition with increasing dosage of CON1. CON1 copy number was not associated with cognitive outcomes measured from the Stanford Binet or Raven Matrices. Diagnostic scores were moderately correlated with CON1 copy number, exhibiting a Pearson's r of 0.49 and 0.67 in social and communicative domains, respectively. Repetitive behavior score demonstrated a more modest correlation with CON1 copy number, with a Pearson's r of 0.26.

**Table 1:** Results from multivariate regression analyses.

Outcome	beta	SE	p-value
Social Diagnostic Score^a^ ^b^	0.25 point increase per copy increase of CON1	0.11	0.021
Communicative^b^ Diagnostic Score	0.18 point increase per copy increase of CON1	0.08	0.030
Repetitive Behaviors Diagnostic Score^b^	0.10 point increase per copy increase of CON1	0.05	0.047
Standardized Social Score	0.43 point decrease per copy increase of CON1	0.23	0.056

Beta estimates, standard errors (SE) and p-values from multivariate regression controlling for sex, age, head circumference, multiplex/simplex status, Stanford Binet full scale IQ, and the interaction of CON1 and simplex/multiplex status.

a In multiplex children.

b In verbal children.

## Discussion

These findings represent the first evidence indicating that, in individuals with ASD, increasing DUF1220 CON1 dosage is associated with increasing severity of the primary symptoms of ASD. Further, the apparent dosage effect detected here suggests a causal role for DUF1220 in ASD symptoms, as previous variants in the 1q21 region detected in ASD are exceedingly rare and do not exhibit the broad normal distribution displayed by DUF1220 CON1 copy number. While the precise manner by which DUF1220 dosage affects ASD symptom severity is not yet known, the evidence presented here indicates that DUF1220 protein domains (specifically clade CON1) have an ASD-wide effect and, as such, are likely to be part of a key pathway underlying ASD severity. Given our recent data linking DUF1220 with neural stem cell proliferation (J. Keeney, submitted), this effect could be related to the timing and rate of neurogenesis, such that too many neurons produced too quickly may result in an overabundance of poorly connected neurons. This initial overabundance would in turn inhibit the formation of long distance projection neurons. This process, resulting from (or exacerbated by) CON1 dosage increase, could in turn lead to the excess of localized versus long-distance connectivity seen in individuals with ASD [17].

The correlation of the dosage of a highly repeated DNA sequence with symptom severity, while new to ASD, has been seen in other cognitive diseases such as Fragile X and Huntington's disease [18]–[20]. However, in contrast to the small size of the repeating unit in those diseases (i.e. 3 nucleotides), the example presented here is the first to link copy number increase of an entire protein domain (approximately 1.7 kb) to disease severity. Also, it is particularly striking that the data presented here, together with our previous findings relating DUF1220 copy number to human brain evolution [10], [15], [16], imply that both expansion of the human brain and increase in autism severity appear to involve increasing dosage of sequences within the same gene family. This intriguing observation may help explain the fact that autism, though maladaptive and heritable, nevertheless persists at a high frequency worldwide.

Our finding that the DUF1220 CON1 copy number spectrum is not demonstrably different between ASD and otherwise healthy individuals suggests that, while DUF1220 CON1 dosage increase contributes to symptom severity in individuals with ASD, an additional contributing factor is needed for disease manifestation. Such factors could include epigenetic effects or other types of previously unexamined genetic variations such as a copy number imbalance among the six DUF1220 clades, both of which represent testable hypotheses for future research. The study also provides evidence that genetic variants that exert significant effects on complex disease phenotypes, such as described here for ASD, can be found in previously unexamined parts of the human genome. Finally, these findings, by implicating the dosage of a previously unexamined, highly copy number polymorphic and brain evolution-related protein domain in ASD severity, provide a major new direction for further research into the genetic factors underlying ASD.

## Materials and Methods

### Ethics Statement

All participants utilized in this study participated in the Autism Genetic Research Exchange (AGRE) and all data was de-identified. The Colorado Multiple institutional Review Board approved this research.

Using the AGRE database, we selected 170 well-characterized non-Hispanic white unrelated individuals with idiopathic autism as subjects for this study (Table 2). AGRE is an academic genetic repository containing genetic material and extensive phenotype information from individuals with autism and unaffected family members [21]. Individuals utilized from the AGRE database were clinically identified utilizing the Autism Diagnostic Interview–Revised (ADI-R) and the Autism Diagnostic Observation Schedule (ADOS). All non-idiopathic forms of autism such as fragile X were excluded from this study. Simplex and multiplex status was also collected due to previous reports suggesting different symptoms and different etiologies depending on familial status [22]. Simplex families are defined in AGRE as those with either a single affected child with an unaffected sibling, or one set of affected identical (monozygotic) twins with an unaffected sibling. Multiplex families are defined as those with more than one affected child (except for one set of monozygotic twins, as noted). Additionally, raw head circumference was collected as a potential confound due to the link between head circumference and autism-like symptoms [5] and the link between CON1 copy number and head circumference [10]. Sex and age were also collected for adjustment purposes. Finally, a control population of 25 healthy non-Hispanic white male individuals was utilized to explore DUF1220 copy number differences between individuals with ASD and otherwise healthy individuals. All DNA samples, including those from unaffected individuals, were collected and prepared from cell lines by the Rutgers branch of the AGRE repository.

**Table 2:** 

Population Characteristics (n = 170)	Proportion or Range and (mean)
Proportion Male	82%
Age years	1.7–30.6 (9.8)
Proportion Multiplex	52%
Age of First Word months	6–108 (24.7)
Social Diagnostic Score	6–30 (21.2)
Communicative Diagnostic Score	5–26 (17.1)
Repetitive Behaviors Diagnostic Score	0–12 (6.9)
VABS Standardized Social Score	23–105 (65.7)
Stanford Binet Full Scale IQ	40–128 (89.7)
Stanford Binet Verbal IQ	43–139 (87.7)
Stanford Binet Non Verbal IQ	42–128 (92.7)
Raven Matrices IQ	28–143 (101.3)

Characteristics related to ASD were measured by common diagnostic and assessment tools including the ADOS, ADI-R, Vineland Adaptive Behavior Scales (VABS), Raven Progressive Matrixes (RM), and the Stanford-Binet Intelligence Scales (SB). The ADOS is a clinician administered, structured-play diagnostic exam designed to evaluate the core symptoms of autism. The ADOS has 5 versions that are administered to the child's developmental ability regardless of age. Due to the age independence of this assessment, deriving severity from the ADOS is non-trivial. Therefore, this study used the ADOS only as an enrollment mechanism, dropping children with a negative autism ADOS indication. The ADI-R is a 2–3 hour parent interview administered by a trained clinician focused on a thorough developmental history and specific behaviors associated with the core symptoms of ASD. ADI-R Social Diagnostic Score, Communicative Diagnostic Score, and Repetitive Behavior Diagnostic score were used as outcomes in this analysis. Importantly, sub-domain scores of the ADI-R have been used quantitatively [5], [23] and higher scores on a diagnostic algorithm indicate greater symptom manifestation. The VABS is a parent questionnaire that addresses the child's personal skills. It is widely used in children with various neurodevelopmental conditions to assess adaptive functioning in social, communication, daily living, and motor skills. The VABS Social Score, Daily Living Score, and Motor Skills Score were used in this study, with lower scores indicating a greater impairment. The RM are multiple-choice tests of abstract reasoning that rely primarily on pattern recognition and are considered good measures of non-verbal abstract abilities. The SB is a commonly used, psychometrically validated measure of intellectual functioning. Verbal (VIQ) and Non-Verbal IQ (NVIQ) measures were used in this analysis.

Droplet digital polymerase chain reaction (ddPCR), a third-generation PCR protocol was utilized following the manufacturer's protocol to assess CON1 copy number in each individual. Primer sequences were as follows: CON1: Left – ‘AATGTGCCATCACTTGTTCAAATAG’, Right – ‘GACTTTGTCTTCCTCAAATGTGATTTT’, Hyb – ‘CATGGCCCTTATGACTCCAACCAGCC’; RPP30 (reference sequence): Left – ‘GATTTGGACCTGCGAGCG’, Right – ‘GCGGCTGTCTCCACAAGT’, Hyb – ‘TTCTGACCTGAAGGCTCTGCGC’. Each sample was run in triplicate to confirm results and the copy number estimates were then merged to produce a final copy number for each sample. The ddPCR assay was found to be highly reproducible (Pearson's r = 0.87–0.97, and ICC>0.75). Importantly, all samples were assayed in a blinded and randomized order. Blinding and randomization of samples guarded against biases by eliminating differential misclassification and as such the results presented are likely underestimates. Randomization is a critical step in this study because it ensures the error due to imperfect measurement is not disproportionately distributed among individuals.

Multivariate linear regression was then utilized to test associations of CON1 with the behavioral phenotypes described. Linear regression was utilized due to the normal distributions of the psychometric outcomes described and due to the normal distribution of CON1 (Figure 1). Diagnostic analyses did not identify outlying or highly leveraged residuals. In all models covariates were explored because of their known or suspected association with autism-like symptoms and/or potential association with CON1 copy number. These included: sex, age, SB IQ (in the case of autism symptoms measured from the ADI-R and VABS), head circumference, multiplex/simplex status and the interaction of CON1 copy number with multiplex/simplex status. We hypothesized that the interaction of CON1 by multiplex/simplex status could be important due to reports suggesting different symptoms, and potentially different etiologies based on this classification [22]. Interactions of CON1 by sex were similarly explored due to increased prevalence of ASD identified in males [1]. A p-value of less than 0.05 was used for definition of significance for main effects. While interactions of CON1 by sex were not significant, the interaction of CON1 by multiplex/simplex approached significance (p = 0.088) in the ADI-R Social Diagnostic Score analysis. Given this finding, subsequent ADI-R Social Diagnostic Score analyses were stratified and results are presented from multiplex individuals. Prior to stratification CON1 copy number was associated with ADI-R Social Diagnostic Score (p = 0.020).
