# Correction: DUF1220 Dosage Is Linearly Associated with Increasing Severity of the Three Primary Symptoms of Autism

**DOI:** 10.1371/journal.pgen.1004373

**Published:** 2014-04-11

**Authors:** The PLOS Genetics Staff

In the Results section the last two sentences are incorrect. The corrected sentences can be seen below.

Incorrect sentences:

Diagnostic scores were moderately correlated with CON1 copy number, exhibiting a Pearson's r of 0.49 and 0.67 in social and communicative domains, respectively. Repetitive behavior score demonstrated a more modest correlation with CON1 copy number, with a Pearson's r of 0.26.

Corrected sentences:

As might be expected diagnostic scores were moderately correlated with each other, ranging from a Pearson′s r of 0.49 to 0.67 in social and communicative domains on the measures utilized. However repetitive behavior score was not as correlated with social and communicative domains ranging from Pearson′s r of 0.26 to 0.55.
